# Vacancy Engineering of Selenium-Vacant NiCo_2_Se_4_ with Enhanced Electrochemical Performance for Supercapacitor

**DOI:** 10.3390/molecules29194580

**Published:** 2024-09-26

**Authors:** Jianjian Fu, Lei Li, Qian Xue, Lindong Li, Zhiying Guo, Lanxiang Meng, Changwei Lai, Yao Guo

**Affiliations:** 1 Henan Joint International Research Laboratory of Nanocomposite Sensing Materials, School of Materials Science and Engineering, Anyang Institute of Technology, Anyang 455000, China; jianjianfu@ayit.edu.cn (J.F.); xueqian418@outlook.com (Q.X.); menglx@ayit.edu.cn (L.M.); laichangwei0229@163.com (C.L.); 2 Key Laboratory for Special Functional Materials of Ministry of Education, Henan University, Kaifeng 475004, China; 3 School of Physics and Electrical Engineering, Anyang Normal University, Anyang 455000, China; 4 School of Chemical and Environmental Engineering, Anyang Institute of Technology, Anyang 455000, China; lilindong-1985@163.com (L.L.); shfu1986@126.com (Z.G.)

**Keywords:** vacancy engineering, NiCo_2_Se_4_, supercapacitor, electrochemical properties

## Abstract

Vacancy engineering effectively modulates the electronic properties of electrode materials, thereby improving their electrochemical performance. In this study, we prepared selenium-deficient NiCo_2_Se_4_ (Se_v_-NCS) using ethylene glycol as a reducing agent in NaOH alkaline environment, and investigated its potential as an electrode material for supercapacitors. Both theoretical and experimental results confirmed that the introduction of vacancies altered the morphology and electronic structure of NiCo_2_Se_4_, which in turn synergistically improved the conductivity and the diffusion capability of electrolyte ions. The optimized Se_v_-NCS electrode achieved an excellent specific capacitance of 2962.7 F g^−1^ at a current density of 1 A g^−1^ and superior cycling stability with a capacitance retention of 89.5% even after 10,000 cycles. Furthermore, an asymmetric device composed of the optimized Se_v_-NCS electrode as the positive electrode and activated carbon as the negative electrode achieved an energy density of 55.6 Wh kg^−1^ at a power density of 800 W kg^−1^. Therefore, this work offers novel insights into the role of vacancy engineering in improving the performance of transition metal compound-based electrode materials for supercapacitor.

## 1. Introduction

Continuous energy consumption and environmental issues have driven significant research into renewable energy and novel energy-storage devices [1,2,3]. Among these, supercapacitors have emerged as promising candidates due to their high power densities, long cycle life, and high charge/discharge rates [4,5,6]. According to their charge-storage mechanism, supercapacitors can be classified into electric double-layer capacitors (EDLCs) and Faradaic capacitors [7,8,9]. EDLC-based carbonaceous materials possess high electrical conductivity and power density. However, their inferior energy densities hamper their practical applications. In contrast, Faradaic capacitors utilizing redox-active materials provide high specific capacitance and adequate energy density owing to the redox reactions involved during the charge/discharge processes [10,11,12].

Recently, extensive efforts have been dedicated to transition-metal selenides that exhibit electrochemical properties, which are comparable to those of transition-metal oxides and sulfides [13,14,15,16]. These selenides offer improved conductivity due to their metallic characteristics. Bimetallic selenides, in particular, show high theoretical capacitance and enhanced electrochemical activity, thanks to the synergistic effects of the bonds between different metal cations [17,18]. Despite these promising properties, the gap between actual and theoretical capacities remains, primarily due to insufficient control over their microstructure. This limitation reduces the diffusion of electrolyte ions and effective surface area, thereby hindering redox reactions and overall charge storage capacity [19,20]. Therefore, optimizing electrode microstructure is critical to improving electrochemical performance.

Vacancy engineering has emerged as a promising strategy to enhance the electrochemical performance of electrode materials [21,22,23,24]. This method introduces lattice defects, distortions, and dislocations, which in turn modify the physical and chemical properties of the material. Vacancies significantly alter the electronic structure and surface characteristics of the electrode, enhancing ion diffusion and electron transfer, which boosts overall performance [25,26]. To date, various synthesis methods, including heat [27,28], plasma [21,29], and chemical reduction treatment [30,31], have been applied to create anion vacancies. However, extreme conditions could lead to excessive vacancies, disrupting the chemical composition of the electrode and the crystal structure, ultimately compromising its electrochemical performance [32]. Therefore, controlling the vacancy concentration is essential for optimal results.

In this study, a moderately green solvent, ethylene glycol (EG)/NaOH, was used as a mild reducing agent to generate adjustable anion vacancies. The optimized Se-vacant NiCo_2_Se_4_ (Se_v_-NCS) with controllable concentrations exhibited improved electrochemical kinetics. Additionally, the optimized electrode demonstrated an impressive specific capacitance of 2962.7 F g^−1^ at a current density of 1 A g^−1^, along with outstanding cycling stability, retaining 89.5% of its capacitance after 10,000 cycles. An asymmetric device with Se_v_-NCS as the positive electrode and activated carbon (AC) as the negative electrode achieved an energy density of 55.6 Wh kg^−1^ at a power density of 800 W kg^−1^. These findings highlight the potential of vacancy control in developing advanced electrode materials for energy-storage devices.

## 2. Results and Discussion

**Figure 1:**
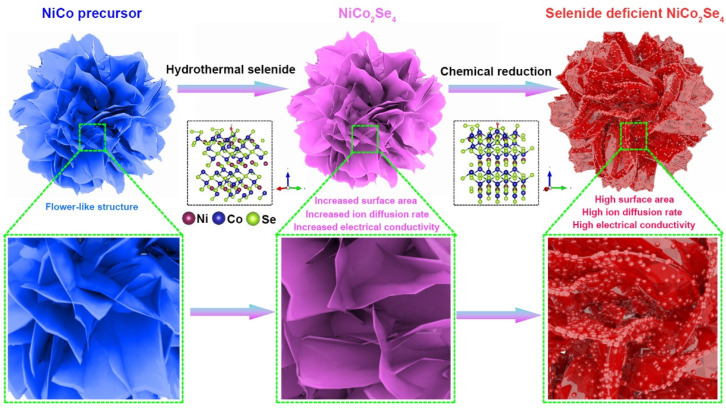
Schematic illustration for the synthesis of selenium-vacant NiCo_2_Se_4_.

Figure 1 illustrates the preparation process for selenium-deficient NiCo_2_Se_4_ (Se_v_-NCS). First, we prepared the NiCo precursor through a conventional hydrothermal reaction method, as depicted in previous studies [33]. During this process, Ni^2+^ and Co^2+^ undergo hydrolysis and self-assemble through continuous nucleation and crystal growth. We used scanning electron microscopy (SEM) to reveal the microstructure of the sample and found that the precursor retained a flower-like structure, composed of multiple interlaced nanosheets with smooth surfaces, as shown in Figure S1. The diffraction pattern of the precursor can be well indexed to Ni(OH)_2_ (JCPDS 38-0715) and Co(OH)_2_ (JCPDS 51-1731), as shown in Figure S2. The results of XRD analysis confirmed that the crystalline phase of the precursor aligns with previously reported layered double hydroxides (LDHs) [34,35]. Next, we used Na_2_SeO_3_ as selenium source to convert the NiCo precursor into NiCo_2_Se_4_ through a secondary hydrothermal reaction. SEM analysis in Figure S3 shows that after selenization, the microstructure of NiCo_2_Se_4_ closely resembles that of the NiCo precursor. Further observations revealed that the surface of the nanosheets became rougher, confirming the occurrence of the selenization reaction. Finally, we prepared selenium-deficient NiCo_2_Se_4_ using ethylene glycol as a reducing agent in NaOH alkaline environment. Ethylene glycol, a mild reducing agent, allows for the deformation of anion vacancies without altering the crystal structure or morphology of the electrode materials [36,37].

**Figure 2:**
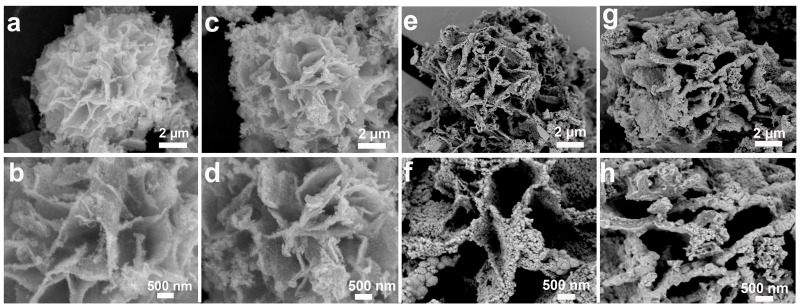
Low- and high-magnification SEM images of the (**a**,**b**) Se_v_-NCS-1, (**c**,**d**) Se_v_-NCS-2, (**e**,**f**) Se_v_-NCS-3, (**g**,**h**) Se_v_-NCS-4.

To study the effect of the reduction reaction, we studied the effect of different concentrations of EG/NaOH solution on the morphology of the microstructure, as shown in Figure 2. After 8 h reaction (Se_v_-NCS-1), SEM images reveal sparse nanoparticles on the surface of the nanosheets (Figure 2a,b). After 12 h, the Se_v_-NCS-2 exhibited interconnected nanosheets with corrugated surfaces (Figure 2c,d). These corrugations will increase the specific surface area, provide additional redox reactions sites, and create effective pathways for electrolyte ion transport. After 16 h (Se_v_-NCS-3), the corrugations begin to aggregate (Figure 2e,f). When the reaction time reached ~18 h (as shown in Figure 2g,h), for the Se_v_-NCS-4 sample, these corrugations aggregated further, resulting in a reduction in the surface area, hindering the permeability of electrolyte ions and limiting the electrode material usage efficiency. Since Se_v_-NCS-2 demonstrated the best electrochemical performance, we conducted in-depth characterization studies on this sample.

**Figure 3:**
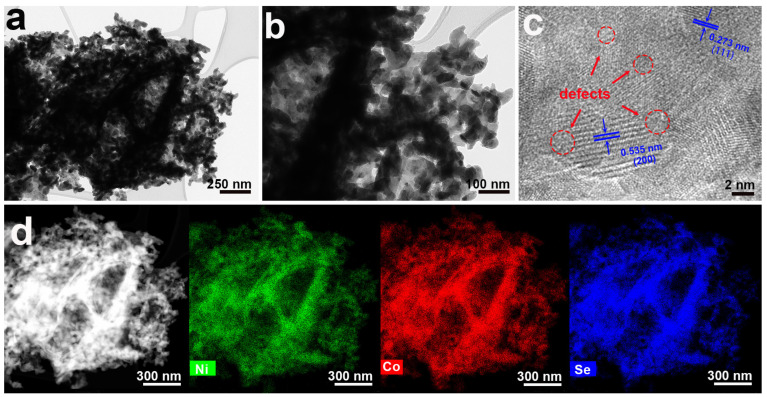
(**a**,**b**) TEM images and (**c**) HRTEM image of the Se_v_-NCS-2 (red circles is the defects). (**d**) Elemental mapping images of the Ni, Co, and Se elements in the Se_v_-NCS-2 sample.

The microstructure of the Se_v_-NCS-2 was further examined through transition electron microscopy (TEM) images (Figure 3). The low-resolution TEM images were consistent with SEM images, showing that Se_v_-NCS-2 had a flower-like structure and the nanosheets consisted of numerous nanopores (Figure 3a,b). The high-resolution TEM image (HR-TEM) in Figure 3c shows clear lattice fringes with d-spacing of 0.273 nm and 0.535 nm, which can be ascribed to the (111) and (200) planes of the NiCo_2_Se_4_ [38]. These results match well with the XRD data. Compared with the HRTEM of the pristine NiCo_2_Se_4_ (Figure S4), the lattice fringes in the selenium-deficient NiCo_2_Se_4_, highlighted in the red dashed region, become less defined, suggesting lower crystallinity of the selenium-deficient NiCo_2_Se_4_. This phenomenon can be attributed to the presence of vacancies causing distortions and defects within the crystal structure [26,39]. EDX demonstrated that Ni, Co, and Se elements were evenly distributed throughout the structure (Figure 3d).

**Figure 4:**
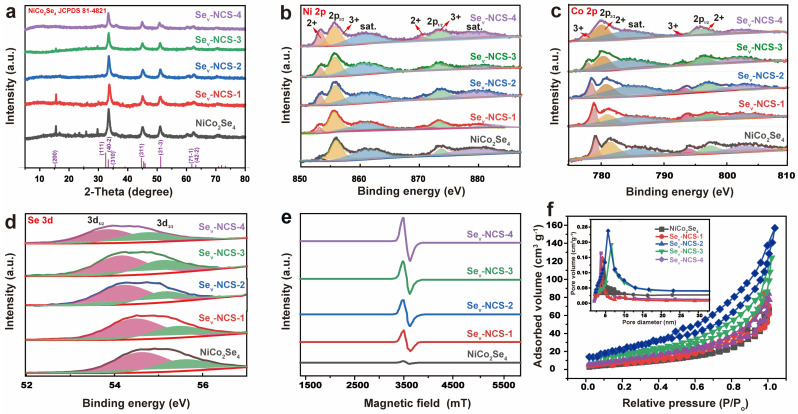
(**a**) XRD pattern and high-resolution of the (**b**) Ni 2p, (**c**) Co 2p, and (**d**) Se 3d XPS spectra in the NiCo_2_Se_4_ and Sev-NCS-*n (n* = 1, 2, 3, and 4) samples. (**e**) EPR curves, (**f**) N_2_ adsorption–desorption isotherm, and the inset of the (**f**) shows the pore size distribution results of the NiCo_2_Se_4_ and Sev-NCS-*n* (*n* = 1, 2, 3, and 4) samples.

Figure 4a show the XRD patterns of the NiCo_2_Se_4_ samples. After the selenide process, all the peaks can be indexed to the NiCo_2_Se_4_ (JCPDS 81-4821) [38,40]. After the vacant engineering process, these samples show similar diffraction pattern with the NiCo_2_Se_4_, suggesting no new phases were introduced. As the reductant concentration increases, the intensity of the peaks become weaker, suggesting clear reduction in crystallinity of the electrode materials [41]. In particular, these weak peaks such as (200) cannot be easily detected as the crystallinity of the material weakens. This phenomenon may be due to the disturbance of lattice structure caused by Se vacancy. In order to estimate the surface chemistry of the samples, the XPS technique was employed. Figure S5 shows the full survey of the NiCo_2_Se_4_, suggesting the presence of the Ni, Co, Se, and O elements. Table S1 shows the element content of the NiCo_2_Se_4_ and Se_v_-NCS-*n* (*n* = 1, 2, 3, and 4) estimated by the XPS analyses, demonstrating slight changes in elemental content after the chemical reduction process. In the high-resolution XPS spectrum of Ni 2p (Figure 4b), the Ni 2p_3/2_ can be deconvoluted into one spin-orbital doublets at binding energy of 852.7 and 855.3 eV, along with one shake-up satellite at 860.7 eV [42]. The Ni 2p_1/2_ can be fitted with one spin-orbital doublets at binding energy of 870.3 and 873.1 eV, along with one shake-up satellite at 879.5 eV [43]. The peaks with binding energy at 855.3 and 873.1 eV can be assigned to Ni^3+^ and peaks with binding energy at 852.7 and 870.3 eV corresponding to Ni^2+^. Compared to NiCo_2_Se_4_, the Ni 2p peak of the Se_v_-NCS-*n* (*n* = 1, 2, 3, and 4) shifted to a lower binding energy and the atomic ratio of the Ni^2+^/Ni^3+^ gradually increased with a higher concentration of EG, suggesting partial conversion of Ni^3+^ to Ni^2+^ [44]. As shown in Figure 4c, the Co 2p spectrum can be divided into two spin-orbital doublets (Co 2p_1/2_ and Co 2p_3/2_) and related satellite [45,46]. The peaks of Co 2p_1/2_ located 796.3 eV and Co 2p_3/2_ located 780.4 eV can be assigned Co^2+^. The peaks of Co 2p_1/2_ located 792.7 eV and Co 2p_3/2_ located 777.7 eV are the characteristic of Co^3+^. Similar with the Ni 2p, the peaks shift positive and the atomic ratio of the Co^2+^/Co^3+^ was gradually increase owing to the transition of the Co^3+^ to Co^2+^ [47]. In Figure 4d, the Se 3d spectrum can be deconvoluted into three peaks, in which two peaks located at 54.5 and 53.6 eV represent Se 3d_3/2_ and Se 3d_5/2_, while another peak centered at 58.5 eV reveals the presence of SeO*_x_* [42]. Based on the XPS spectra of Se 3d, it can be inferred that the binding energies of Se 3d move towards lower values with increasing EG concentration. These negative shifts are related to the generation of Se vacancy through the reduction process of the EG, owing to the generation of surface Se vacancies. These shifts are balanced by the partial conversion of Ni^3+^ to Ni^2+^, as shown in Figure 4b, facilitating electron transfer between the electrode materials [24]. As shown in Figure S6, Raman spectroscopy was also conducted to study the effect of the Se vacancy. For pristine NiCo_2_Se_4_, the peak located at 175 cm^−1^ is related with Ni-Se or Co-Se bonds [48]. Upon the introduction of vacancies, the peak became weaker and shifted to the right, suggesting changes in the crystal structure. As shown in Figure 4e, the electron paramagnetic resonance (EPR) analyses were performed to check the states of the unpaired electron of the NiCo_2_Se_4_ and Se_v_-NCS-*n* (*n* = 1, 2, 3, and 4). For pristine NiCo_2_Se_4_, there is no detectable EPR signal, suggesting the absence of selenide vacancies. After reduction, an evident symmetrical EPR signal related to selenide vacancies, with a g value of 2.003, emerged [49]. Especially, the intensity of the EPR signal increased progressively from Se_v_-NCS-1 to Se_v_-NCS-4, suggesting a rise in vacancy concentration. The generated vacancies increased lattice disorder and the number of unpaired electrons in the material [50].

N_2_ adsorption-desorption was performed to evaluate the BET surface area and pore size distribution of the samples (Figure 4f). A typical type-IV and the hysteresis loop at P/P_0_ range of 0.5~1.0 can be observed, suggesting a mesoporous structure [51]. As result, Se_v_-NCS-2 exhibited the highest BET surface area of 57.4 m^2^ g^−1^, compared to those of NiCo_2_Se_4_, Se_v_-NCS-1, Se_v_-NCS-3 and Se_v_-NCS-4, with surface areas of 43.8, 47.9, 51.6, and 50.1 m^2^ g^−1^, respectively. A higher specific surface area exposes more active sites, which enhances charge storage and redox reactions. The inset of Figure 4f shows all samples have mesopores ranging from 5 to 10 nm. Generally, the Se_v_-NCS-2 electrode, with a high specific surface area and a relatively large mesoporous structure, facilitates rapid ion transport at the electrode/electrolyte interface, leading to improved specific capacitance [52].

**Figure 5:**
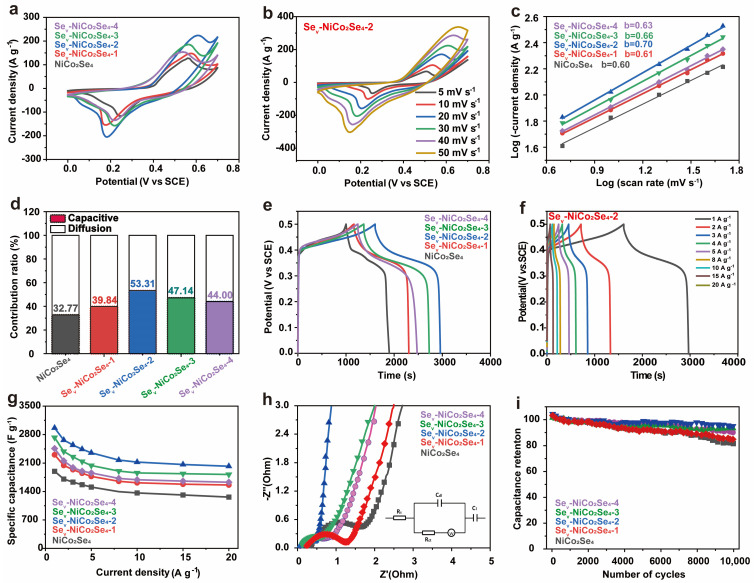
(**a**) Comparative CV curves of the NiCo_2_Se_4_ and Se_v_-NCS-*n* (*n* = 1, 2, 3, and 4) electrodes at a scan rate of 30 mV s^−1^. (**b**) CV curves of Se_v_-NCS-2 at scan rates ranging from 5 to 50 mV s^−1^. (**c**) b values determined from the plot of log(*i*)~log(*v*) for NiCo_2_Se_4_ and Se_v_-NCS-*n* (*n* = 1, 2, 3, and 4) electrodes. (**d**) Percentage contribution of the capacitive and diffusion-controlled processes of NiCo_2_Se_4_ and Se_v_-NCS-n (n = 1, 2, 3, and 4) at scan rate of 10 mV s^−1^. (**e**) Comparative GCD curves of NiCo_2_Se_4_ and Se_v_-NCS-*n* (*n* = 1, 2, 3, and 4) electrodes at a current density of 1 A g^−1^. (**f**) GCD curves of Se_v_-NCS-2 at current densities from 1 to 20 A g^−1^. (**g**) Comparison the specific capacities of the NiCo_2_Se_4_ and Se_v_-NCS-*n* (*n* = 1, 2, 3, and 4) electrodes at current density from 1 to 20 A g^−1^. (**h**) Nyquist plots of the NiCo_2_Se_4_ and Se_v_-NCS-*n* (*n* = 1, 2, 3, and 4) electrodes (inset is the equivalent circuit diagram). (**i**) Cycling performance of NiCo_2_Se_4_ and Se_v_-NCS-*n* (*n* = 1, 2, 3, and 4) electrodes at a current density of 15 A g^−1^.

The electrochemical performance of the electrodes was evaluated in 3 M KOH electrolyte using a three-electrode system. Figure 5a shows the comparative CV curves of all the samples, highlighting obvious redox behavior with cathodic/anodic peak, which come from the reversible Faradaic redox reactions of the OH^–^ in alkaline electrolyte, suggesting the battery-like behavior of the electrode, as shown in the equations below:

NiCo_2_Se_4_ + OH^−^ + H_2_O ↔ NiSeOH + 2CoSeOH + e^−^ (1)

CoSe_2_O_4_ + OH^−^ ↔ CoSeO + H_2_O+ e^−^ (2)

Notably, as the EG volume increases, the peak value and integral area of the oxidation-reduction peak in cyclic voltammetry (CV) first initially rise and then decline. The Se_v_-NCS-2 shows a significantly larger CV integral area compared to NiCo_2_Se_4_, Se_v_-NCS-1, Se_v_-NCS-3, and Se_v_-NCS-4, indicating superior electrochemical performance. CV curves under various scan rates ranging from 5 to 50 mVs^−1^ were recorded as shown in Figure 5b and Figure S7. Generally, the shape of the CV curves are well maintained with obvious redox peaks shifting as the scan rate increases, representing good reversibility for all the samples. To gain deeper insights into the electrochemical performance of electrode materials, the electrochemical reaction kinetics was well studied. In general, the charge storage mechanisms include diffusion-controlled and capacitance-controlled processes, which correspond to the double layer and Faradaic reaction at the electrode surface [53].

*i* = a*v*^b^ (3)

Equation (3) describes the relationship between peak current density (*i*) and scan rate (*v*), with a and b as empirical parameters. The value of b defines the type of electrochemical reaction: b value near 0.5 indicates a diffusion-controlled reaction. While a value of 1.0 suggest a capacitance-controlled process [54,55]. As shown in Figure 5c, the b values of the NiCo_2_Se_4_, Se_v_-NCS-1, Se_v_-NCS-2, and Se_v_-NCS-4 electrode are 0.60, 0.61, 0.69, 0.65, and 0.63 respectively. These values indicate that both surface Faraday redox reaction and ion intercalation are involved during the charge/discharge process. Additionally, according to *i* = k_1_*v* + k_2_*v*^0.5^, (where k_1_ and k_2_ are constants at a given potential) represents the currents generated by capacitance-controlled and diffusion-controlled processes, respectively [56]. We conducted a quantitative analysis of the capacitance-controlled and diffusion-controlled contributions to the electrode performance, as shown in Figure 5d and highlighted in the dark cyan region of Figure S8. At a scan rate of 10 mV s^−1^_,_ the capacitance contribution to the Se_v_-NCS-2 electrode was about 53.31%. The capacitance contributions for NiCo_2_Se_4_, Se_v_-NCS-1, Se_v_-NCS-3 and Se_v_-NCS-4 electrodes were 32.77%, 39.84%, 47.14%, and 44.00%, respectively. These results highlight the beneficial role of selenide vacancies in enhancing reaction kinetics. The improved capacitance may be attributed to the increased electrolyte contact at the electrode surface and the faster charge transfer due to Se vacancies. As shown in Figure S9, when the scanning rate is increased from 5 to 50 mV s^−1^, the capacitance contribution ratio of the electrode increases further verifies the rapid reaction kinetics characteristics of the electrodes.

The comparative GCD profiles of the NiCo_2_Se_4_, Se_v_-NCS-1, Se_v_-NCS-2, and Se_v_-NCS-4 electrode at a current density of 1 A g^−1^ are shown in Figure 5e. As expected, the Se_v_-NCS-2 possess longer charge/discharge time and higher specific capacitance, which are consistent with the CV results. The distinctive plateaus of GCD plot suggest the presence of the redox reaction during the charge/discharge process. The GCD profile for all samples at various current densities are shown in Figure 5f and Figure S10. The well-maintained shape of the GCD profiles suggests reversible electrochemical behavior of the electrodes. The decrease in specific capacitance at higher current densities is due to ineffective contact between the electrode and electrolyte. The specific capacitance at different current densities is derived from the GCD profiles, as shown in Figure 5g. As a result, the specific capacitance of the NiCo_2_Se_4_, Se_v_-NCS-1, Se_v_-NCS-2, Se_v_-NCS-3 and Se_v_-NCS-4 electrodes at current density of 1 A g^−1^ are 1899.2, 2305.6, 2962.7, 2725.7 and 2459.9 F g^−1^, respectively. With the current density to 20 A g^−1^, the capacitance retention of the Se_v_-NCS-2 are about 68.3%, which are much higher than that of 66.5% (NiCo_2_Se_4_), 67.7% (Se_v_-NCS-1), 66.7% (Se_v_-NCS-3), and 66.2% (Se_v_-NCS-4). As shown in Figure 5h, EIS analyses were applied to investigate the electrochemical conductivity and charge transfer kinetic of the electrode. Typically, the intercept of the plot at the *x*-axis corresponds with the intrinsic resistance (R_s_) of the current collector and electrode materials, the resistance of the electrolyte, and the connect resistance between the electrode and electrolyte. The intersection of the semicircle with the *x*-axis represents charge transfer resistance (R_ct_). The slope of the plot are related to the ion diffusion ability [57]. As a result, the R_s_ and R_ct_ of the Se_v_-NCS-2 are about 0.038 and 0.46 Ω, which are lower than those of counterparts, suggesting the excellent electrical conductivity of the Se_v_-NCS-2, the detail values are shown in Table S2. Additionally, the Sev-NCS-2 electrode shows a steeper slope in the low-frequency region compared to the others, further confirming its enhanced electrochemical performance. Cycling performance of electrodes were performed to investigate the electrochemical stability of the electrode. As shown in Figure 5i, after 10,000 repeat charge/discharge cycle at current density of 20 A g^−1^. The Se_v_-NCS-2 electrode possess a favorable cycling stability with 89.5% retention, corresponding to the NiCo_2_Se_4_ (87.22%), Se_v_-NCS-1 (87.2%), Se_v_-NCS-3 (84.02%), and Se_v_-NCS-4 (75%).

**Figure 6:**
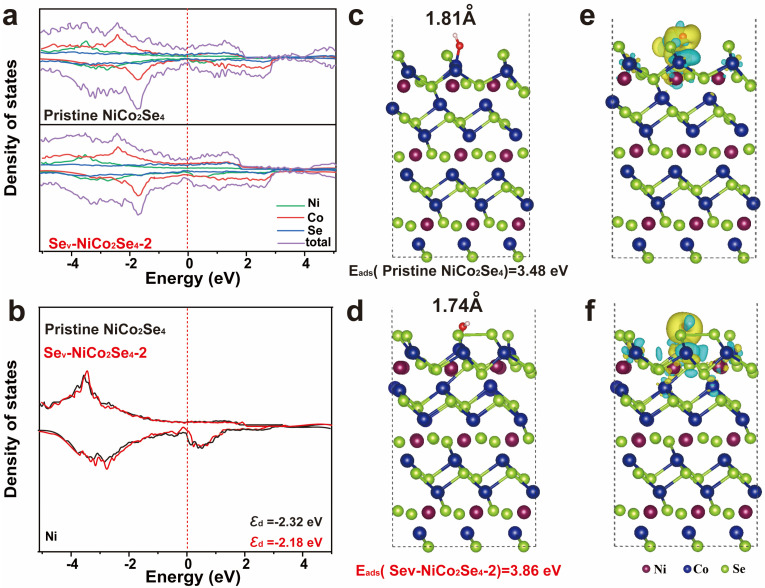
(**a**) Total densities of states and (**b**) the density state of the Ni atom calculated for the pristine NiCo_2_Se_4_ and Se_v_-NCS, the Fermi level is set to 0 eV; the side view and the Eads (OH^−^) value in the (100) slab of (**c**) pristine NiCo_2_Se_4_ and (**d**) Se_v_-NCS. The differences in charge density of the (**e**) NiCo_2_Se_4_ and (**f**) Se_v_-NCS. Where Ni appears in dark purple, Co in dark blue, Se in light green, O in red, and H in white.

To gain more insight into the potential mechanisms of electrochemical performance in Se-vacancy NiCo_2_Se_4_, this study employed density functional theory (DFT) calculations to optimize the adsorption behavior of OH^−^ on both pristine and Se-vacancy NiCo_2_Se_4_ (002) lattice plane. The relevant results are presented in Figure 6a. The increase in density of states near the Fermi level indicates a higher concentration or denser distribution of electronic states. This is typically associated with an enhancement of the electrical conductivity. As shown in Figure 6b, the integration of the Projected Density of States (PDOS) curves reveals that the D-band center of the Ni site in Se_v_-NCS-2 (ε_d_ = −2.18) is closer to the Fermi level compared to the corresponding site in NiCo_2_Se_4_ (ε_d_ = −2.32). According to the D-band center theory, the proximity of the center of the d band to the Fermi level indicates that more electrons are adsorbed. The higher the number of electrons, the better the activity of the electrode materials [58]. Therefore, we can conclude that in Se_v_-NCS-2, the Ni site is more likely to interact with OH^−^ compared to the Ni site in NiCo_2_Se_4_, thereby facilitating electrochemical redox reaction. To further elucidate the impact of interfacial behavior and vacancy defects on electrochemical performance, we conducted calculations of OH^-^ adsorption energy (E_ads_) and the corresponding ion diffusion barriers, as illustrated in Figure 6c,d. It is evident that the E_ads_ value of the Se_v_-NCS-2 (3.86 eV) is significantly higher than that of NiCo_2_Se_4_ (3.48 eV). These results suggest that the creation of abundant vacancies, due to unsaturated dangling bonds and the removal of Se sites greatly enhances OH adsorption capacity. Correspondingly, The OH-Co bond length in Sev-Ncs-2 is approximately 1.74Å, shorter than that inNiCo_2_Se_4_ (1.81 Å), indicating stronger OH^−^ adsorption in Se_v_-NCS-2. The comparison of charge density betweenNiCo_2_Se_4_ and Se_v_-NCS-2 further confirms the strong OH adsorption ability, as shown in Figure 6e,f. Compared to NiCo_2_Se_4_, Se_v_-NCS-2 shows more pronounced charge exchange between the Co nucleus and OH^−^, indicating enhanced electron transfer dynamics. This emphasizes the beneficial effect of introducing vacancies, aligning well with experimental findings. In summary, introducing defects alters the material’s band structure, enhancing electron transport and electrical conductivity. Additionally, defects introduce anisotropy without altering the lattice structure, creating more pathways for ion migration and promoting charge transfer. Moreover, these defects generate unsaturated bonds and new electrochemically active sites, altering electron distribution and enhancing electrochemical activity, thereby improving the material’s overall performance.

**Figure 7:**
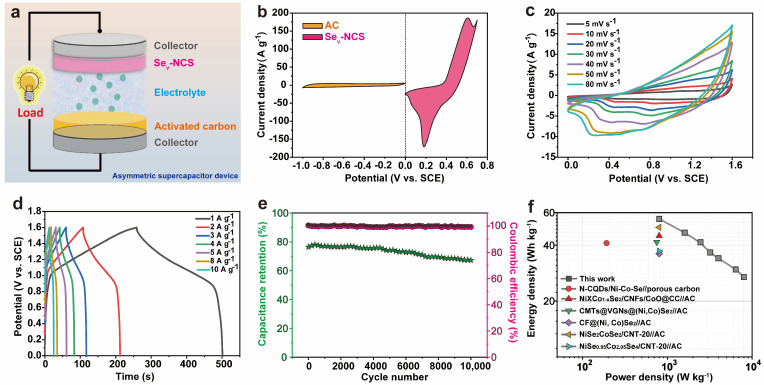
(**a**) Schematic diagram of the as-fabricated Se_v_-NCS-2//AC ASC device. (**b**) CV curves of AC and NiCo_2_Se_4_ at a scan rate of 10 mV s^−1^. (**c**) CV curves at scan rates ranging from 10–100 mV s^−1^. (**d**) GCD curves at current densities of 1~10 A g^−1^; (**e**) cycling stability and coulombic efficiency at a current density of 8 A g^−1^. (**f**) Ragone plots.

To investigate the practical application of the electrode, Se_v_-NCS-2//AC ACS device was constructed as depicted in Figure 7a. The electrochemical performance of the AC electrode was also conducted as shown in Figure S11. Figure S11a shows the CV curves of the AC with rectangular shape at potential window of −1~0 V under various scan rates, Figure S11b exhibit the GCD curves with triangular shape at potential window of −1~0 V under various current densities. These data suggest the EDLC properties of the AC electrode. Figure S11c shows the calculated specific capacitance of the AC. Figure S11d shows the cycling performance of the AC electrode. These results show that the AC has excellent performance and can be used as negative electrode to assemble Se_v_-NCS-2//AC ACS device. To achieve the optimized electrochemical performance, the mass ratio of is carefully estimated according to charge balance theory, which can be expressed q^+^ = q^−^. As shown in Figure 7b, the CV curves of the AC and Se_v_-NCS-2 at 30 mV s^−1^, it can be calculated that the mass ratio of the Se_v_-NCS-2 and AC is about 1:3. The CV curves of the optimized devices under various scan rate at a voltage window of 1.6V were recorded as shown in Figure 7c, the quasi-rectangular shape with reversible redox peaks suggest the combination characteristic of the EDLC and battery-like behavior of the device. With the increase of the scan rates, the shape of the CV curves are well kept, indicating good reversibility of the device. The GCD profiles under various current densities are shown in Figure 7d. The plateaus of the GCD curves represent the existence of the redox reaction, which agree well with the CV result. Subsequently, the specific capacitance of the device can be obtained according to GCD curves as shown in Figure S11. The device possesses specific capacitance of 156.3, 131.6, 109.7, 102.9, 95.8, 83.3, and 75.8 F g^−1^ at current density of 1, 2, 3, 4, 5, 8, and 10 A g^−1^, respectively. The cycling performance was conducted at current density of 8 A g^−1^. As revealed by the 10,000 repeated GCD process in Figure 7e, the device maintained nearly 88.7% of the initial capacitance, representing a high cycling stability. Furthermore, the device exhibited 100% coulombic efficiency after the cycling test, suggesting high electrochemical reversibility. Based on the GCD results under various current densities, the energy density and power densities of the device and the comparative reported literature presented via Ragone plots (Figure 7f). The device shows energy densities of 55.6 Wh kg^−1^ at a power density of 800 W kg^−1^ and the energy density is about 27.0 Wh kg^−1^ at power density of 8000 W kg^−1^. As shown in Table S3, these values are superior than those of other Ni-Co-Se based devices, such as Ni_x_Co_1−x_Se_2_/CNFs/CoO@CC//AC (45 Wh kg^−1^, 800 W kg^−1^) [59], (N-CQDs/Ni-Co-Se//C (41.1 Wh kg^−1^, 191.5 W kg^−1^) [60], CMTs@VGNs@(Ni, Co)Se_2_//AC (41.6 Wh kg^−1^, 750 W kg^−1^) [61], CF@(Ni, Co)Se_2_//AC (36.02 Wh kg^−1^, 800 W kg^−1^) [62], NiSe_2_/CoSe_2_/CNT-20//AC (50 Wh kg^−1^, 800 W kg^−1^) [63], Ni_0.95_Co_2.05_Se_4_//AC (37.22 Wh kg^−1^, 800 W kg^−1^) [64].

## 3. Experiment Details

### 3.1. Preparation of the NiCo_2_Se_4_

The NiCo_2_Se_4_ was prepared through two-step hydrothermal process, as depicted in our previous work [40]. Initially, 0.5 mmol Ni(NO_3_)_2_·6H_2_O and 1 mmol Co(NO_3_)_2_·6H_2_O were dissolved in 30 mL of deionized (DI) water and ethanol mixture solution (volume ratio of 2:1). Then, 0.75 mmol hexamethylenetetramine (HMTA) was added to obtain a pink and homogeneous solution. After magnetic stirring for 30 min, the homogeneous solution was transferred into a Teflon-lined stainless-steel autoclave and then maintained at 100 °C for 12 h. After cooling down to room temperature, the solution was washed with DI water and ethanol. The as-obtained precipitate was collected through centrifugation and dried at 60 °C overnight. After that, 50 mg of the resulted powder, 150 mg of Na_2_SeO_3_ and 5 mL of N_2_H_4_ was dissolved in 25 mL DI water, then the mixture was transferred into a Teflon-lined stainless-steel autoclave and kept at 180 °C for 8 h. After this, the final product was rinsed with DI water and ethanol, and then vacuum-dried at 60 °C overnight.

### 3.2. Preparation of the Defective Engineering of Se_v_-NCS-n (n = 1, 2, 3, and 4)

The vacancy engineering of NiCo_2_Se_4_ was prepared through reduction treatment process to create the Se vacancy. In general, the as-prepared NiCo_2_Se_4_ powders were immersed into a certain amount of 30 mL ethylene glycol (EG) and 30 mmol NaOH solution. The solution was then transferred into the Teflon-lined stainless-steel autoclave and kept at 120 °C for 8, 12, 16, 18 h, respectively. Accordingly, the resulting samples were denoted as Se_v_-NCS-*n* (*n* = 1, 2, 3, and 4), respectively. Subsequently, the final powders were repeatedly rinsed with DI water and ethanol, and finally the products were obtained after dried in the vacuum oven at 60 °C for 12 h.

### 3.3. Characterization of Materials

The crystal structure was characterized by powder X-ray diffraction pattern (XRD, Bruker, Billerica, MA, USA, Advanced D8). The morphology was collected through Field Emission scanning electron microscopy (SEM, Hitachis-4800 operate voltage ~15 kV, Tokyo, Japan) equipped with energy dispersive X-ray spectrum and transmission electron microscopy (TEM, Talos Fx200, New York, NY, USA, accelerate voltage ~200 kV) equipped with selected area electron diffraction (SAED). The chemical status was acquired by X-ray photoelectron spectroscopy (XPS, Thermofisher, Waltham, MA, USA, Escalab 250). The specific surface area and pore size distribution were collected through Nitrogen adsorption/desorption equipment (Micromeritics, Norcross, GA, USA, ASAP2460) using Brunauer–Emmett–Teller (BET) and Barrett–Joyner–Halenda (BJH) method. The unpaired electron spin states of the samples after the introduction of the vacancy were analyzed through electron paramagnetic resonance (EPR) spectrometer (Bruker EMXplus).

### 3.4. Theoretical Computational Details

All calculations based on first-principles were conducted utilizing density-functional-theory (DFT) as implemented in the Vienna ab initio simulation package (VASP, version 5.4.4) [65,66]. The core electron states were addressed using a plane wave projector enhanced wave (PAW) pseudo-potential [67]. The exchange-correlation effect was characterized using the conventional Perdew–Burke–Ernzerhof (PBE) [68] scheme within the framework of the generalized gradient approximation (GGA). The plane-wave energy cut-off was established at 500 eV, accompanied by a Gaussian smearing [69] width of 0.1 eV. The Monkhorst–Pack k-point grids with an interval of 0.2 Å^−1^, were employed for Brillouin zone sampling. A vacuum height of 15 Å along the *z*-axis was implemented to prevent artificial interactions from the periodic images. The self-consistent convergence tolerances for energy and force were set to be 1.0 × 10^−7^ eV and 0.005 eVÅ^−1^, respectively.

The adsorption energy can be confirmed as

*E*_ads_ = *E*_suface_ + *E*_adsorbate_ − *E*_total_ (4)

where *E*_suface_, *E*_absorbate_, and *E*_total_ denote the total energy of surface, adsorbate, and adsorbate on surface, separately. Positive (negative) values indicate that the adsorption is an endothermic (exothermic) reaction.

### 3.5. Electrochemical Tests

The electrochemical properties were investigated through cyclic voltammetry (CV), galvanostatic charge/discharge (GCD), and electrochemical impedance spectroscopic (EIS) through electrochemical workstation (IVIUM) in 3 M KOH aqueous electrolyte at room temperature. The EIS tests were conducted under stable open-circuit voltage, with amplitude of 5 mV and testing frequency range of 100 kHz to 0.01 Hz. The electrochemical test includes three-electrode and two-electrode system. For the three-electrode system, the as-prepared electrode served as working electrode, the HgO electrode served as reference electrode. The Pt foil served as counter electrode. The working electrode were fabricated through mixing the active powder, black carbon, and polytetrafluoroethylene (PTFE) with a weight ratio of 8:1:1 to form a slurry. The slurry was then pasted on nickel foam current collector with area of 1 × 1 cm^2^ and mass loading of 3 mg cm^−2^ and dried at 60 °C overnight.

The specific capacitance C_s_ (F g^–1^) or specific capacity Q_s_ (C g^–1^) was estimated according to the GCD curves using Equations (5) and (6):

(5) $$C_{s}=\frac{I\Delta t}{m\Delta V}$$

(6) $$Q_{s}=\frac{I\Delta t}{m}$$

where I (mA), m (mg), ΔV (V), and Δt (s) signify the applied current, mass loading of active materials, potential window, and discharge time, respectively.

For the two-electrode system, the asymmetric supercapacitor (ACS) device was assembled using Se_v_-NCS as positive electrode, AC as negative electrode, and 3 M KOH aqueous electrolyte. Due to the different charge storage capacities of the positive and negative electrodes of asymmetrical supercapacitors, it is necessary to match the charges of the positive and negative electrodes based on the principle of charge balance equation Q^+^ = Q^–^. The mass ratio of the positive and negative electrodes was determined according to the following formula:

*m*^+^*C*^+^*V*^+^ = *m*^−^*C*^−^*V*^−^ (7)

where m^+^ (mg), C^+^ (F g^−1^), and V^+^ (V) represent the mass, specific capacitance, and potential window of the positive electrode, m^−^ (mg), C^−^ (F g^−1^), and V^−^ (V) denote the mass, specific capacitance, and potential window of AC, respectively.

The specific capacitance (C_d_, F g^−1^), energy density (E, W h kg^−1^), power density (P, W kg^−1^) and coulombic efficiency (*η*) of the ASC device were calculated using the following equations:

(8) $$C_{d}=\frac{I\times \Delta t}{M\times \Delta V}$$

(9) $$E=\frac{1}{2}C_{d}\Delta V^{2}$$

(10) $$P=\frac{E}{\Delta t}$$

(11) $$\eta =\frac{C_{d}}{C_{c}}\times 100\%$$

where I (A), Δt (s), M (g), ΔV (V), and C_c_ (F g^−1^) represent the discharge current, discharge time, total mass of active materials of the positive and negative electrodes, potential window of the device, and specific capacitance of the ACS device, respectively.

## 4. Conclusions

In conclusion, a series of selenium-deficient NiCo_2_Se_4_ materials were successfully synthesized using a facile hydrothermal method combined with a reduction process using ethylene glycol as a reducing agent in NaOH alkaline environment. The optimized Se_v_-NCS-2 exhibited remarkable electrochemical properties as a supercapacitor electrode, achieving a specific capacitance of 2962.7 F g^−1^ at a current density of 1 A g^−1^ and cycling life of (89.5% capacitance retention after 10,000 cycles). Furthermore, the asymmetric Se_v_-NCS-2//AC ACS device achieved an energy density of 55.6 Wh kg^−1^ at a power density of 800 W kg^−1^. Therefore, NiCo_2_Se_4_ with sufficient Se vacancies shows great potential for application in energy storage devices.

## Data Availability

Data are contained within the article and Supplementary Materials.

## Supplementary Materials

The following supporting information can be downloaded at: https://www.mdpi.com/article/10.3390/molecules29194580/s1. Refs. [40,59,60,61,62,63,64,70,71,72,73,74] are cited in Supplementary Materials.
