# Supplementary material for: Vacancy Engineering of Selenium-Vacant NiCo2Se4 with Enhanced Electrochemical Performance for Supercapacitor
Source: Molecules. 2024 Sep 26;29(19):4580. doi: 10.3390/molecules29194580 (PMC11478223; doi:10.3390/molecules29194580)
Supplement: Supplementary file 1 [file molecules-29-04580-s001.zip › molecules-3174317-supplementary.pdf]

Supporting Information

Vacancy Engineering of Selenium-Vacant $NiCo_2Se_4$ with Enhanced Electrochemical Performance for Supercapacitor

Jianjian Fu [1,2], Lei Li [3,*], Qian Xue [1], Lindong Li [4], Zhiying Guo [4], Lanxiang Meng [1], Changwei Lai [1] and Yao Guo [1,*]

[1] Henan Joint International Research Laboratory of Nanocomposite Sensing Materials, School of Materials Science and Engineering, Anyang Institute of Technology, Anyang 455000, China; jianjianfu@ayit.edu.cn (J.F.); xueqian418@outlook.com (Q.X.); menglx@ayit.edu.cn (L.M.); laichangwei0229@163.com (C.L.)

[2] Key Laboratory for Special Functional Materials of Ministry of Education, Henan University, Kaifeng 475004, China

[3] School of Physics and Electrical Engineering, Anyang Normal University, Anyang 455000, China

[4] School of Chemical and Environmental Engineering, Anyang Institute of Technology, Anyang 455000, China; lilindong-1985@163.com (L.L.); shfu1986@126.com (Z.G.)

* Correspondence: kaomu2@aynu.edu.cn (L.L.); guoyao@ayit.edu.cn (Y.G.)

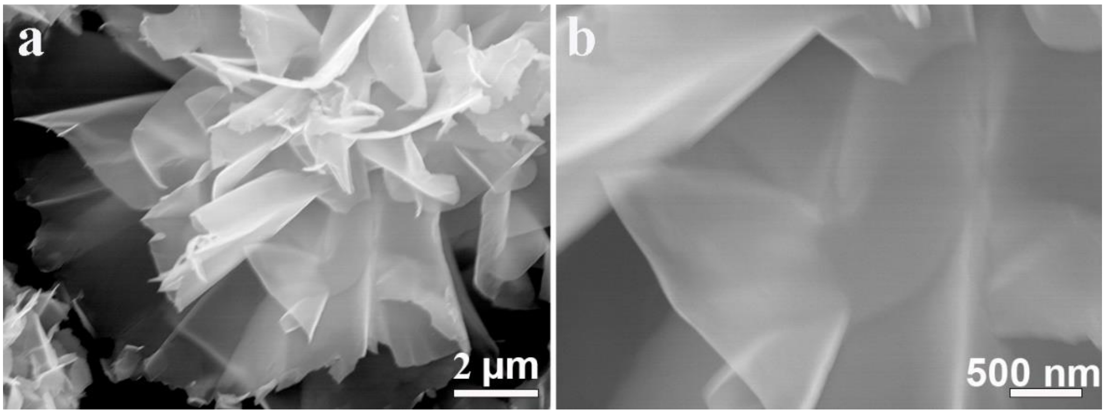

Figure S1. SEM images of the NiCo precursor under different magnification.

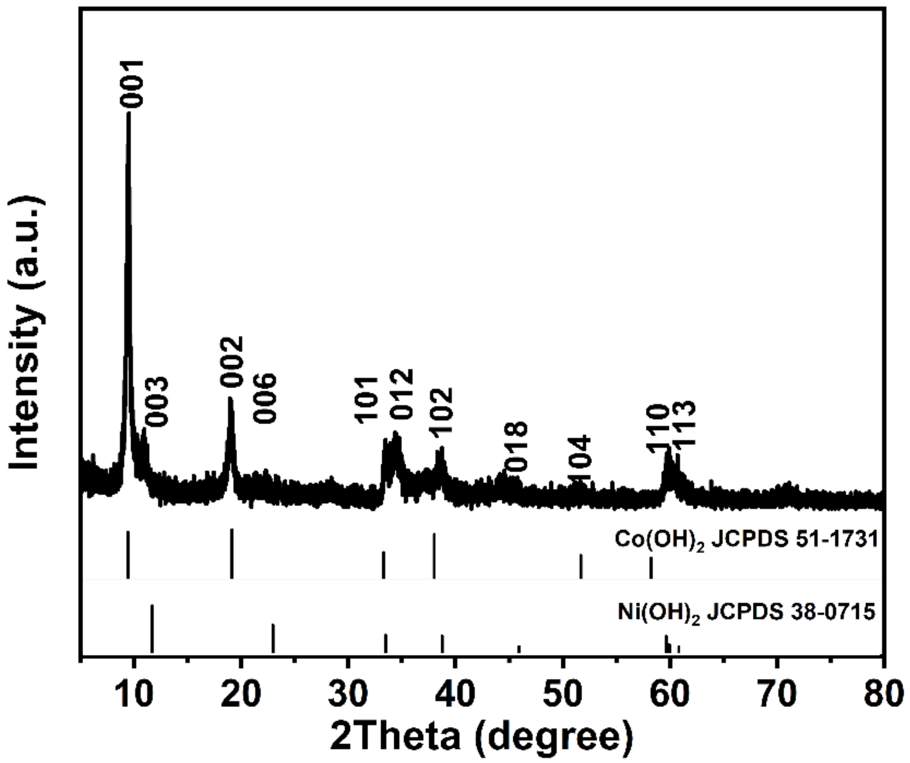

Figure S2. XRD pattern of the NiCo precursor.

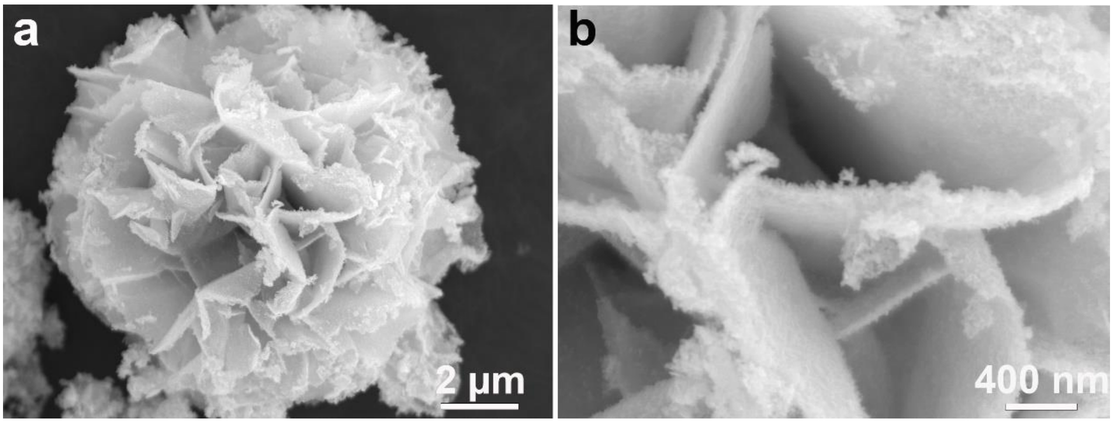

Figure S3. SEM images of the $NiCo_2Se_4$ under different magnification.

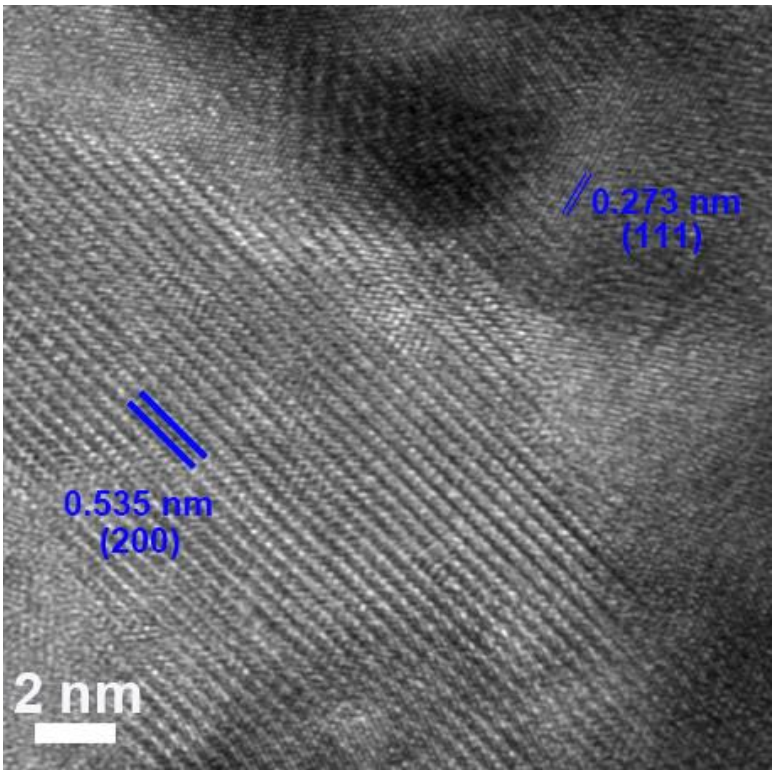

Figure S4. HRTEM of the pristine $NiCo_2Se_4$.

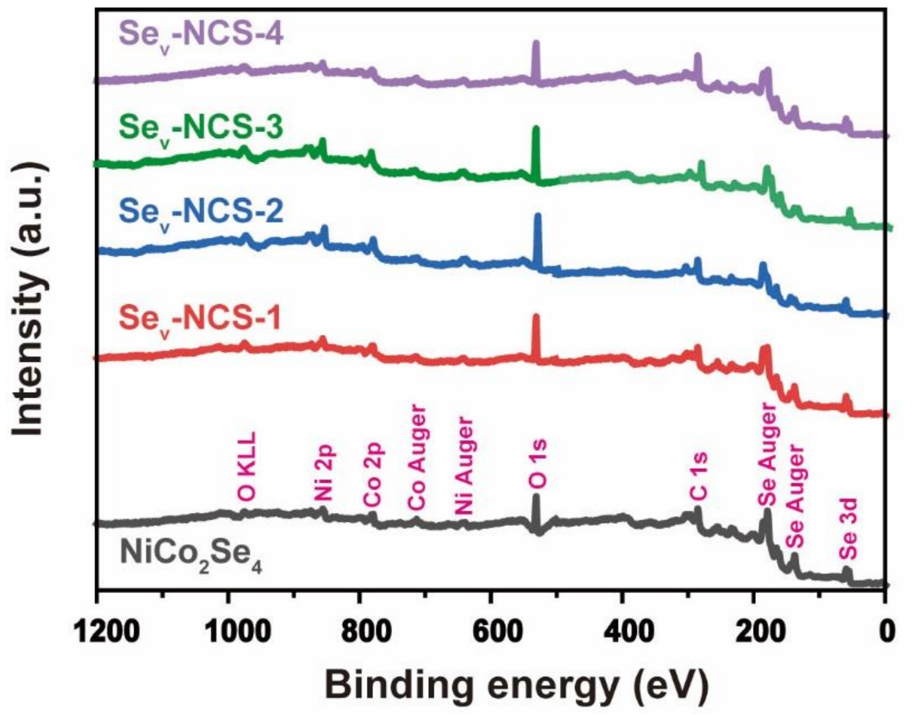

Figure S5. Total XPS survey of the $NiCo_2Se_4$ and Sev-NCS-n (n = 1, 2, 3, and 4).

Table S1 Element content of the $NiCo_2Se_4$ and Sev-NCS-n (n = 1, 2, 3, and 4) estimated by the XPS analyzes

	**$NiCo_2Se_4$**	**Se_v-NCS-1**	**Se_v-NCS-2**	**Se_v-NCS-3**	**Se_v-NCS-4**
**Ni(at.%)**	14.35	14.69	16.11	16.50	17.28
**Co(at.%)**	30.66	33.85	34.83	35.71	36.97
**Se(at.%)**	54.99	51.46	49.06	47.79	45.75

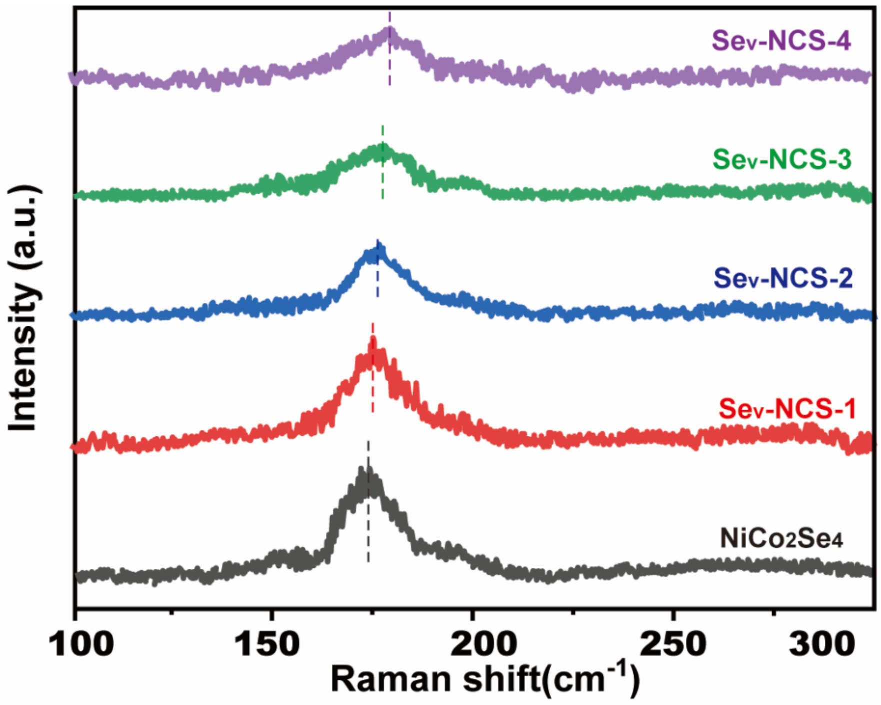

Figure S6. Comparative Raman spectrum of the $NiCo_2Se_4$ and Sev-NCS-n (n = 1, 2, 3, and 4).

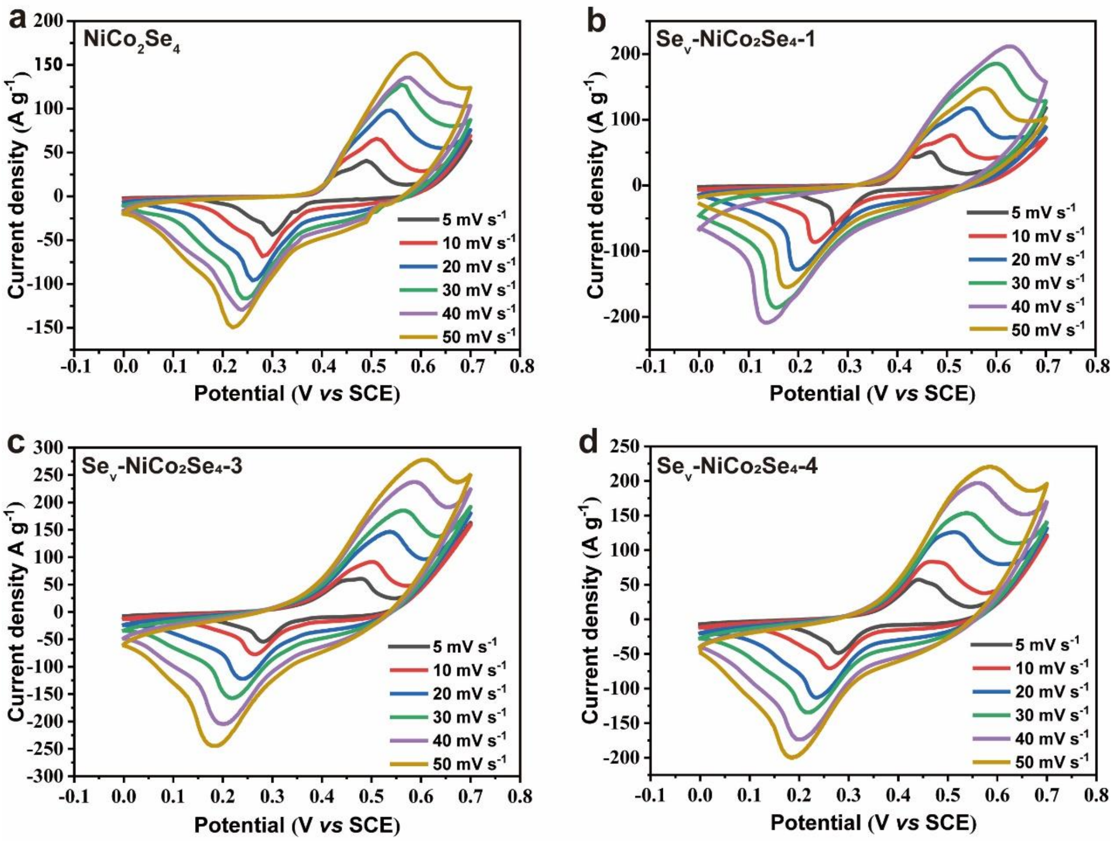

Figure S7. CV curves of the (a) $NiCo_2Se_4$, (b) Se_v-NCS-1, (c) Se_v-NCS-3, (d) Se_v-NCS-4 at scan rate range from 5 to 50 mV s^{-1}.

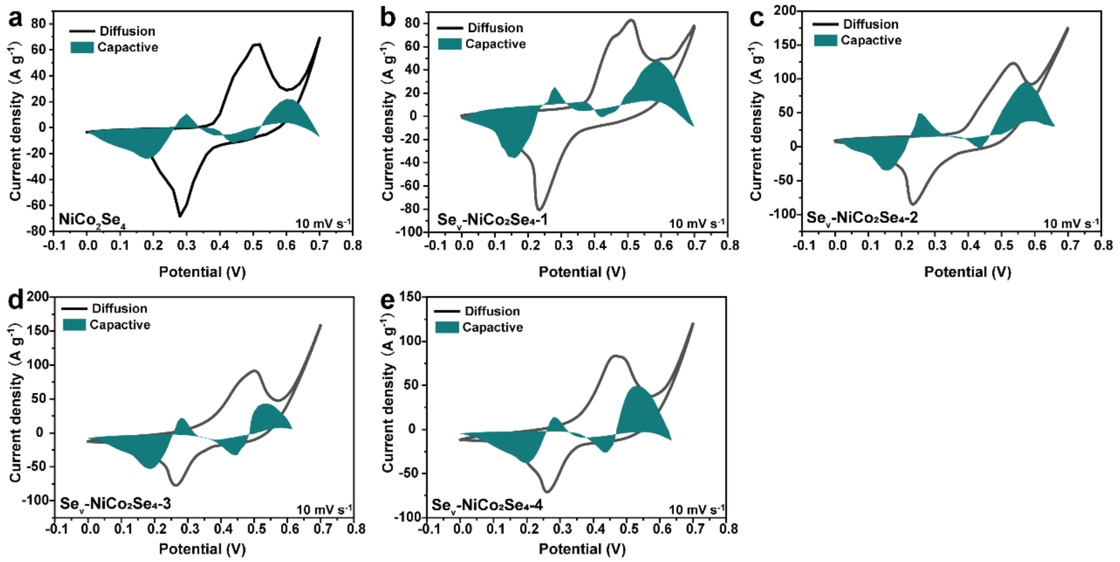

Figure S8. The capacitive contribution (dark cyan shaded area) of the (a) $NiCo_2Se_4$, (b) Se_v-NCS-1, (c) Se_v-NCS-2, (d) Se_v-NCS-3, (e) Se_v-NCS-4 in the CV curves at scan rate of 10 mV s^{-1}.

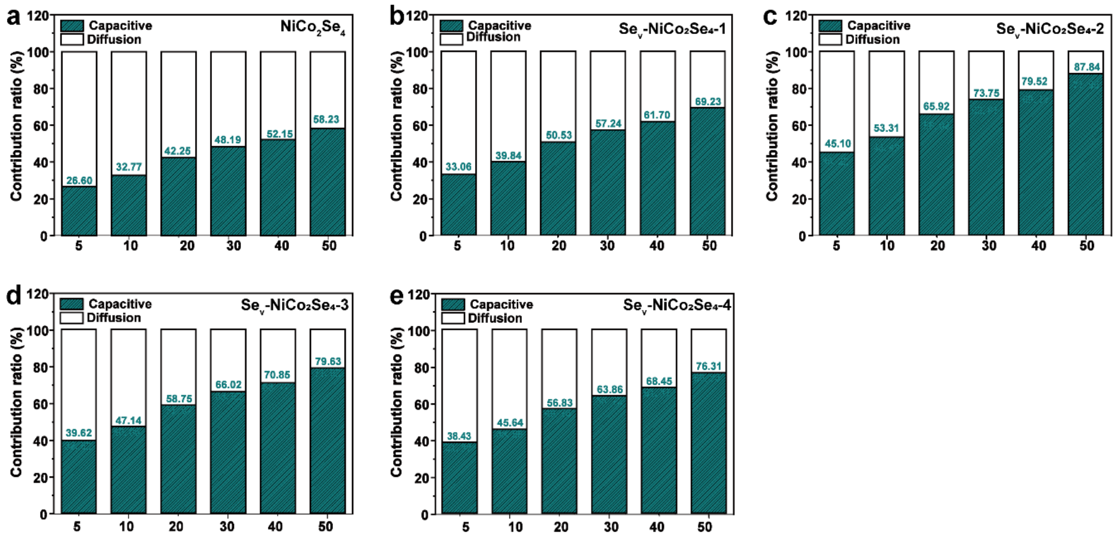

Figure S9. The contribution ratio of the capacitive and diffusion contribution of the (a) $NiCo_2Se_4$, (b) Se_v-NCS-1, (c) Se_v-NCS-2, (d) Se_v-NCS-3, (e) Se_v-NCS-4 in the CV curves at scan rate of 5, 10, 20, 30, 40, 50 mV s^{-1}.

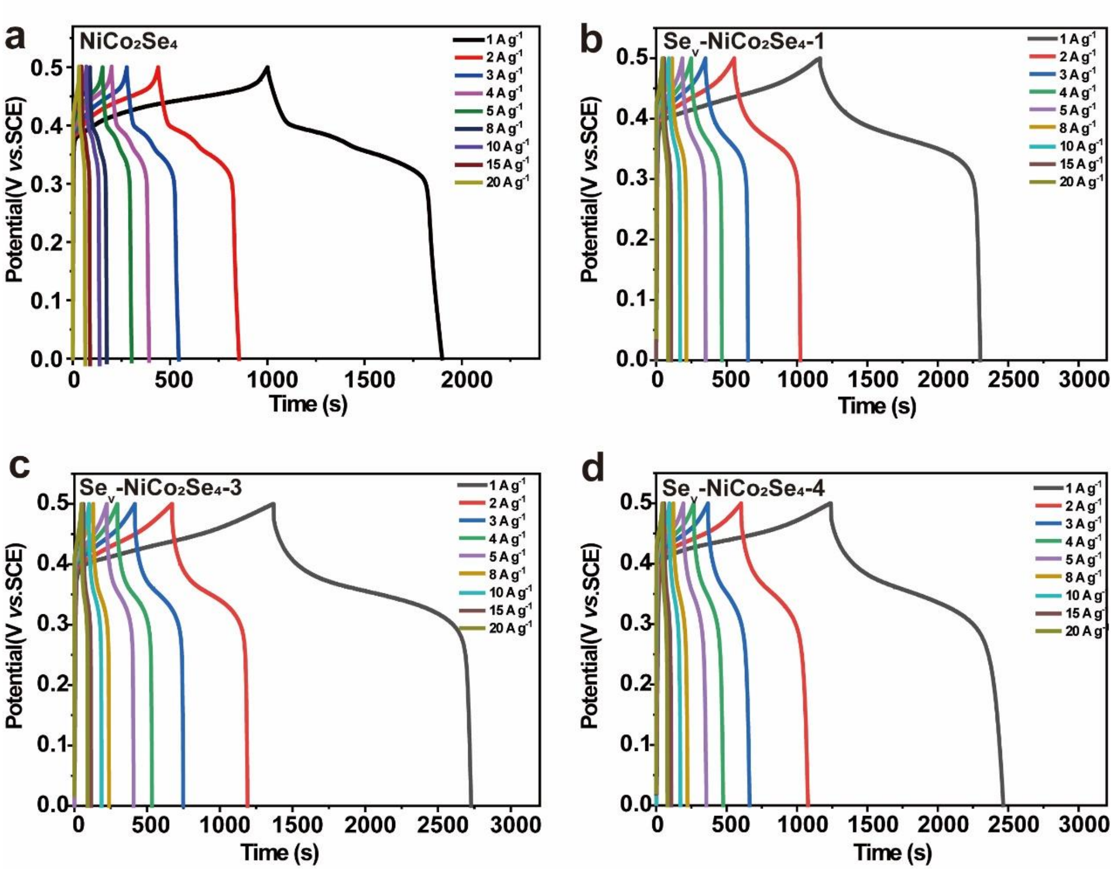

Figure S10. The comparative GCD curves of the (a) $NiCo_2Se_4$, (b) Se_v-NCS-1, (c) Se_v-NCS-3, (d) Se_v-NCS-4 at current densities of 1 ~ 20 A g^{-1}.

Table S2 The fitting values of the EIS using Zview software

	$NiCo_2Se_4$	Se_v-NCS-1	Se_v-NCS-2	Se_v-NCS-3	Se_v-NCS-4
**R_s (Ω)**	0.329	0.08	0.038	0.09	0.25
**R_{ct} (Ω)**	1.498	0.489	0.46	0.445	0.851

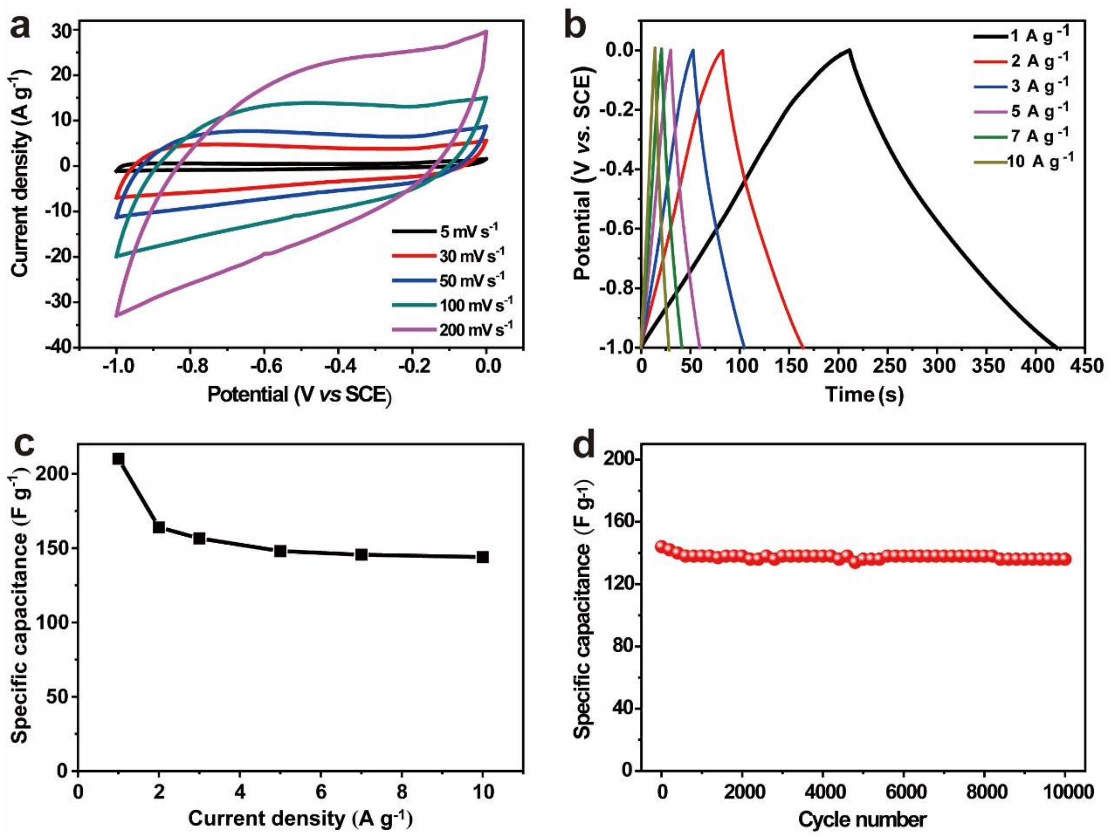

Figure S11. The electrochemical performance of the AC electrode. (a) the CV curves under different scan rate from 5 to 200 mV s^{-1}. (b) the GCD curves under different current densities from 1 to 10 A g^{-1}. (c) the calculated specific capacitance under different current densities from 1 to 10 A g^{-1}. (d) the cycling performance under 10000 repeat charge/discharge process.

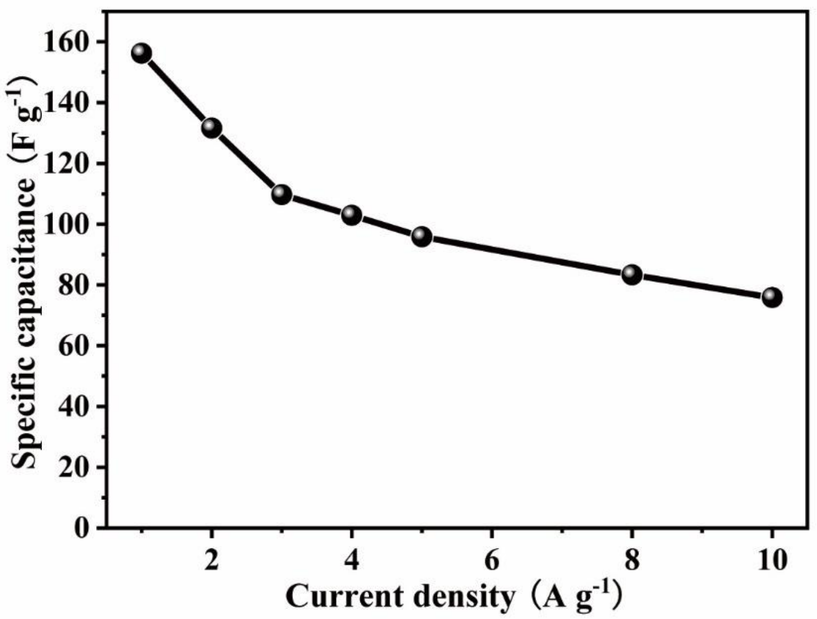

Figure S12. The specific capacitance of the Se_V-NCS-2//AC ACS device under different current densities from 1 to 10 A g^{-1}.

Table S3 Comparison of the energy and power densities of $NiCo_2Se_4$ electrode material supercapacitors in literatures

Materials	Energy density (Wh Kg^{-1})	Power density (W Kg^{-1})	Ref.
**This work**	**55.6**	**800**	**Our work**
$Ni_xCo_{1-x}Se_2$/CNFs/CoO@CC//AC	45	800	[1]
N-CQDs/Ni-Co-Se//C	41.1	191.5	[2]
CMTs@VGNs@(Ni, Co)Se_2//AC	41.6	750	[3]
CF@(Ni, Co)Se_2//AC	36.02	800	[4]
$NiSe_2$/$CoSe_2$/CNT-20//AC	50	800	[5]
$Ni_{0.95}Co_{2.05}Se_4$//AC	37.22	800	[6]
$NiCo_2Se_4$//AC	42.4	1600	[7]
$NiCo_2Se_4$//AC	24.03	1055	[8]
Ni-Co-Se//AC	38.5	802.1	[9]
CoSe//AC	18.6	750	[10]
Ni@$Ni_{0.8}Co_{0.2}Se$//AC	17	1526.8	[11]
$NiCo_2Se_4$/rGO//rGO	37.83	1433.55	[12]
$NiCo_2Se_4$//AC	25	490	[13]

References

1. Sun, L.; Liu, Y.; Yan, M.; Yang, Q.; Liu, X.; Shi, W. Lewis acid etched $NixCo_{1-x}Se_2$ derived from ZIF-L on CoO nanowires for hybrid-supercapacitors. *Chem. Eng. J.* **2022**, *431*, 133472.
2. Lu, Z.; Hu, Z.; Xiao, L.; Xie, Y.; Li, N.; Xi, L.; Chen, W.; Xiao, J.; Zhu, Y. Battery-type Ni-Co-Se hollow microspheres cathode materials enabled by bifunctional N-doped carbon quantum dots with ultrafast electrochemical kinetics for hybrid supercapacitors. *Chem. Eng. J.* **2022**, *450*, 138347.
3. Yin, X.; Han, L.; Fu, Y.; Lu, J.; Song, Q.; Li, H. $(Ni, Co)Se_2$ nanoparticles on vertical graphene nanosheets@carbon microtubes for high-performance solid-state asymmetric supercapacitors. *J. Energy Storage* **2022**, *53*, 105205.
4. Yang, Q.; Liu, Y.; Deng, C.; Sun, L.; Shi, W. In-situ construction of heterostructure $(Ni, Co)Se_2$ nanoarrays derived from cone-like ZIF-L for high-performance hybrid supercapacitors. *J. Colloid Interface Sci.* **2022**, *608*, 3049-3058.
5. Zheng, J.; Bai, X. Preparation of Ni-Co PBA-derived beaded $NiSe_2/CoSe_2$/CNT for high-performance supercapacitors. *J. Alloys Compd.* **2023**, *944*, 169110.
6. Jiang, B.; Liu, Y.; Zhang, J.; Wang, Y.; Zhang, X.; Zhang, R.; Huang, L.-L.; Zhang, D. Synthesis of bimetallic nickel cobalt selenide particles for high-performance hybrid supercapacitors. *RSC advances* **2022**, *12*, 1471-1478.
7. Li, L.; Guo, Y.; Li, L.; Lai, C.; Tang, Z.; Lou, X.; Ju, L.; Fu, J. Self-assembled microflower-like $NiCo_2X_4$ (X= O, S, Se) as electrodes for asymmetric supercapacitors. *J. Alloys Compd.* **2023**, 172913.
8. Guo, Z.; Diao, Y.; Han, X.; Liu, Z.; Ni, Y.; Zhang, L.J.C. Mesoporous $NiCo_2Se_4$ tube as an efficient electrode material with enhanced performance for asymmetric supercapacitor applications. *CrystEngComm* **2021**, *23*, 2099-2112.
9. Qu, G.; Zhang, X.; Xiang, G.; Wei, Y.; Yin, J.; Wang, Z.; Zhang, X.; Xu, X.J. ZIF-67 derived hollow Ni-Co-Se nano-polyhedrons for flexible hybrid supercapacitors with remarkable electrochemical performances. Chinese Chem. Lett. **2020**, *31*, 2007-2012.
10. Zhu, Y.; Huang, Z.; Hu, Z.; Xi, L.; Ji, X.; Liu, Y.J. 3D interconnected ultrathin cobalt selenide nanosheets as cathode materials for hybrid supercapacitors. Electrochim. Acta **2018**, *269*, 30-37.
11. Guo, K.; Cui, S.; Hou, H.; Chen, W.; Mi, L.J. Hierarchical ternary Ni–Co–Se nanowires for high-performance supercapacitor device design. Dalton Tran. **2016**, *45*, 19458-19465.
12. Ghosh, S.; Samanta, P.; Samanta, P.; Murmu, N.C.; Kuila, T.J. Investigation of electrochemical charge storage efficiency of $NiCo_2Se_4$/RGO composites derived at varied duration and its asymmetric supercapacitor device. *Energy & Fuels* **2020**, *34*, 13056-13066.
13. Li, S.; Ruan, Y.; Xie, Q.J. Morphological modulation of $NiCo_2Se_4$ nanotubes through hydrothermal selenization for asymmetric supercapacitor. Electrochim. Acta **2020**, *356*, 136837.
